# Community Stakeholder Perspectives on Children With Cerebral Palsy and Their Caregivers: A Qualitative Study From Rural Malawi

**DOI:** 10.1111/cch.70361

**Published:** 2026-10-06

**Authors:** Bente van der Meijden, Victoria von Salmuth, Alpheus Njewa, Alinafe Chisulanda Kusate, Ria Reis, Wieger Voskuijl

**Affiliations:** ^1^ Amsterdam UMC, Location University of Amsterdam Amsterdam Institute for Global Child Health, Emma Children's Hospital Amsterdam the Netherlands; ^2^ Department of Family Medicine Kamuzu University of Health Sciences Blantyre Malawi; ^3^ Care and Public Health Research Institute, Department of Family Medicine, Universiteitsingel Maastricht University Maastricht the Netherlands; ^4^ Shirati KMT Hospital Mara region Tanzania; ^5^ Department of Physiotherapy Mangochi District Hospital Mangochi Malawi; ^6^ Amsterdam UMC, Location University of Amsterdam, Department of Global Health Amsterdam Institute for Global Health and Development Amsterdam the Netherlands; ^7^ Leiden, the Netherlands and Children's Institute Leiden University Medical Center Cape Town South Africa; ^8^ University of Cape Town Cape Town South Africa; ^9^ Department of Paediatrics and Child Health Kamuzu University of Health Sciences Blantyre Malawi

**Keywords:** cerebral palsy, community health, disability, health promotion, low‐ and middle‐income countries, Malawi

## Abstract

**Background:**

Cerebral palsy (CP) disproportionately affects children in low‐ and middle‐income countries, where stigma and discrimination lead to marginalization and reduced participation. To design culturally appropriate community‐based health promotion strategies that can address these challenges, the role of community stakeholders is crucial, yet unstudied in rural sub‐Saharan Africa. This study aimed to explore stakeholders’ perceptions, beliefs and attitudes towards children with CP and their caregivers in rural Malawi.

**Methods:**

A qualitative study using semistructured focus group discussions was conducted with 68 stakeholders across five villages in Mangochi District, Malawi, from November 2022 to January 2023. Participants included traditional leaders, religious leaders, teachers, health surveillance assistants, traditional healers and community volunteers. Thematic analysis identified key themes regarding knowledge, perceptions, community participation and future needs.

**Results:**

The following four major themes emerged: (1) limited knowledge about CP causes and treatment, with CP often attributed to infectious diseases, witchcraft or pregnancy complications; (2) perception of difficulties and their impact on the children and their caregivers; with financial constraints being named most and problems accessing education; (3) community participation and response; were stigma was identified as primary barrier to health promotion and community participation; and (4) future facilitators to improve quality of life including strong stakeholder willingness to support integrated care approaches.

**Conclusions:**

Community stakeholders demonstrated positive attitudes towards children with CP and willingness to support inclusive care programmes, despite limited disease‐specific knowledge. Findings suggest that community‐based health promotion interventions addressing knowledge gaps, reducing stigma and strengthening referral pathways could significantly improve outcomes for children with CP and their families in low‐resource settings.

## Key Messages

- Community stakeholders in rural Malawi demonstrated significant knowledge gaps about CP, relying on stigmatizing terminology and attributing the condition to witchcraft, infectious diseases and ‘wrongdoing,’ contribution to stigma and social isolation of the affected families.
- Financial constraints, limited educational access and stigma were identified as primary barriers affecting children with CP and their families, with mothers particularly vulnerable to abandonment and social isolation.
- Despite these challenges, community stakeholders demonstrated positive attitudes and strong willingness to support inclusive care programmes, highlighting existing community structures as a meaningful foundation for health promotion
- Stakeholders identified community sensitization, improved healthcare worker training, clearer referral pathways and integrated support addressing both medical and socioeconomic needs as priorities for strengthening care for children with CP.

**Abbreviations**
CP: cerebral palsy
FDGs: focus group discussions
HAS: health surveillance assistant
HIC: high‐income countries
LMIC: low‐ and middle‐income countries
MCV: Malawi Children Village
QoL: quality of life
TA: thematic analysis
WHO: World Health Organization

## 1 Introduction

Cerebral palsy (CP) represents the most common childhood motor disability globally, affecting approximately 50 million people worldwide with a prevalence of 2–2.5 per 1000 live births (Imam et al. 2021). The burden of CP is substantially higher in low‐ and middle‐income countries (LMIC) often attributed to delayed diagnosis, limited access to early intervention services and inadequate rehabilitation programmes (Donald et al. 2014). In Sub‐Saharan Africa (SSA), prevalence may be five to 10 times higher than in high‐income countries HIC (Tataryn et al. 2017).

CP is primarily characterized by abnormalities in movement and posture and is frequently accompanied by comorbidities such as intellectual impairment, feeding difficulties, epilepsy and chronic pain (Bax et al. 2005; Novak et al. 2012). Beyond these clinical challenges, children with CP and their families, are disproportionately affected by stigma, neglect and discrimination, particularly in resource‐limited settings. This results in marginalization and reduced participation in community life (Donald et al. 2014; Lassetter et al. 2007; Gray 2002b; Green 2007). Community perceptions and responses to disability significantly influence the quality of life, social inclusion and developmental outcomes for children with CP and their families (Lassetter et al. 2007; Pretorius and Steadman 2018). Families may also face substantial financial strain, limited educational opportunities and low levels of formal and informal support (Donald et al. 2014; Lassetter et al. 2007; Pretorius and Steadman 2018). Together, these challenges contribute to a reduced quality of life (QOL) compared to peers without CP (Donald et al. 2014; Gray 2002b; Green 2007; Pretorius and Steadman 2018; Mitra et al. 2011) and adversely affect caregivers psychosocial wellbeing highlighting broader psychosocial impact on the whole family (Donald et al. 2014; van der Meijden, Kusate, et al. 2026).

These challenges are particularly pronounced in rural settings where specialized services are even more scarce and community understanding of disability may be limited (Green 2007; Adnams 2010; Sage et al. 2023). As a result, CP in LMICs is a medical condition inextricably linked to social, economic and environmental determinants of health (WHO 2022). Addressing these determinants requires health promotion approaches that go beyond individual education to include community‐level action on stigma, discrimination, isolation and exclusion, while strengthening support systems and meaningful inclusion.

Community stakeholder engagement is recognized as a critical component for sustainable health promotion and rehabilitation interventions for children with disabilities in resource‐limited settings (WHO 2022). However, intervention success depends heavily on community acceptance, understanding and the beliefs and attitudes of those who influence social norms and access to services. Research demonstrates that community attitudes towards disability can either facilitate or hinder the effectiveness of health promotion and rehabilitation efforts (scior 2011). Positive attitudes are associated with improved social integration, educational inclusion and family well‐being, while negative attitudes perpetuate exclusion and limit access to essential services (scior 2011; McNally and Mannan 2013). However, evidence on stakeholder knowledge, beliefs and attitudes towards children with CP remains scarce in SSA, representing a critical gap for the development of contextually grounded and effective health promotion strategies in these settings.

In Malawi, CP is the most common physical disability (Tataryn et al. 2017). The only available national prevalence study reported a CP prevalence of 1.7 per 1000 children (Katangwe‐Chirwa et al. 2026). Importantly, in this study, 90.5% of the identified children were classified at Gross Motor Function Classification System (GMFCS) levels III–IV, strongly suggesting underdetection of less severely affected children. Combined with the fact that this estimate falls well below the global average of 2–2.5 per 1000 children in SSA, the true burden in Malawi is likely substantially greater than reported. Mangochi District, with its dense population of approximately 1.4 million people (Opendataforafrica, n.d.), exemplifies the challenges faced by rural communities with limited specialized services and minimal community‐based support in providing adequate care for children with disabilities (Eide 2003). No district‐level prevalence data currently exists, representing a critical gap for local health planning.

Since 2022, our team has operated the first multidisciplinary (outreach) CP clinics in the district, providing weekly rehabilitation, medical and nutrition services, alongside caregiver education, to better understand their child's condition. So far, it has served over 300 children with CP and their care‐takers. During education sessions, caregivers—especially mothers—consistently describe experiences of stigma, limited support, social isolation and exclusion from community activities. While the caregiver perspective is increasingly documented (van der Meijden, Wedda, et al. 2026; Paget et al. 2015), less is known about how community stakeholders in rural Malawi perceive children with CP and their families and how these perceptions may contribute to or reduce stigma and barriers to inclusion.

Key stakeholders, including traditional leaders, religious figures, educators, healthcare workers and community volunteers, shape community responses to disability through their influence on cultural explanations, social norms and local support structures (Lemmi et al. 2015). Understanding their beliefs about the causes of disability, appropriate care‐seeking and the rights and roles of children with CP can directly affect whether families are supported, included and able to access services (Pitten 2018; Kang‐Yi et al. 2018). Understanding these perspectives is therefore critical for designing culturally appropriate community‐based health promotion strategies that address stigma and discrimination and promote inclusion and caregiver wellbeing.

This study explored the perceptions, beliefs and attitudes of key community stakeholders towards children with CP and their caregivers in rural Malawi. By identifying both barriers and opportunities for community‐based health promotion initiatives, the findings aim to inform the development of locally acceptable health promotion initiatives to reduce stigma, improve inclusion and strengthen support for families affected by CP.

## 2 Materials and Methods

### 2.1 Study Design and Setting

A qualitative study using semistructured focus group discussions (FGDs) was conducted in Mangochi District, southern Malawi, from November 2022 to January 2023. A semistructured approach was used to allow flexibility within the interviews, enabling us to probe deeper into emerging themes while ensuring that key topics were covered consistently across the groups (Braun and Clarke 2021). This study was part of research projects to inform and improve care for children with CP in a multidisciplinary care program, conducted in collaboration with local partners at Mangochi District Hospital and Malawian Children Village (MCV), a local nongovernmental organization.

The research was undertaken during the pilot phase prior to the formal launch of our multidisciplinary outreach cerebral palsy clinic. During this period, caregivers shared their experiences of stigma, limited support and exclusion within their respective communities. As these factors influence the overall health of both the child and the caregiver, we conducted this study to gain insights into community perspectives. Specifically, the study aims to explore how community stakeholders in rural Malawi perceive children with CP and their families and how these perceptions may contribute to or reduce stigma and barriers to inclusion. The findings can inform health promotion strategies and service delivery approaches.

The clinic was subsequently established at MCV's premises, which already operated a rehabilitation program for children with CP. MCV implements various programmes across 30 villages north of Mangochi town, including disability services for children. MCV has community health volunteers who identify children with disabilities and facilitate referrals to our multidisciplinary outreach CP clinic.

Mangochi District was selected due to its high population density (approximately 1.4 million inhabitants) and existing rehabilitation services. Five villages were purposively selected based on having at least five children with CP known to community health volunteers through MCV's outreach programmes: Bolera, Mpemba, Nkungumbe, Chikundo and Njereza. These villages fall within the intended catchment area of the planned clinic services.

### 2.2 Participants and Recruitment

Key stakeholders were defined as community members who were not caregivers of children with CP but held influential positions within their communities. Participants included traditional leaders (chiefs and counsellors), religious leaders (Christian and Muslim), educators (primary and nursing school teachers), community health workers (health surveillance assistants [HSAs] working for the government and community health volunteers from MCV working for a local NGO) and traditional healers.

Recruitment was facilitated through existing collaboration between the research team, MCV staff and HSAs. Village chiefs were contacted through phone initially, and when they confirmed participation, they were asked to invite other stakeholders. Two participants from each stakeholder category per village were invited by the chief to ensure adequate representation.

### 2.3 Data Collection

Semistructured FGDs were conducted in local languages (Chichewa or Chiyao) by a bilingual male Malawian researcher with a background in Anthropology and Education, fluent in both languages. Interviews were held in quiet community spaces (school or community hall), to ensure confidentiality and participant comfort.

The topic guide was theoretically underpinned by the international Classification of Functioning, Disability and Health (ICF), given its established utility in conceptualizing disability across biological, individual and environmental domains (World Health Organization 2001). The guide was developed through literature review and in close collaboration with local partners, with content mapped into ICF domains as followed: knowledge and beliefs about CP, including perceived causes and treatment options (body functions and structures); barriers and facilitators to participation of children and families across relevant community settings—schools, healthcare and religious activities (activity and participation); community responses, stigma, referral pathways and available support (environmental factors); and the impact of CP on individual family members and caregiver wellbeing (personal factors). The topic guide was first pilot‐tested with four stakeholders to refine clarity and flow. No major problems were identified during the pilot; however, several additional prompts were added to the questions to facilitate discussion. The primary researcher (B.v.d.M.) trained the data collector in FGD moderation to ensure consistency during the data collection.

In each community, two separate FDGs were conducted. One with the traditional leaders (i.e., chiefs and counsellors) and one with the other stakeholders (i.e., religious leaders, educators, community health workers and traditional healers) to respect cultural norms and existing community hierarchies that prevent chiefs to express their opinions in the presence of their subjects. One additional interview was conducted exclusively with community health volunteers affiliated with MCV. During all interviews two authors (B.v.d.M. and V.v.S.) were present to observe interactions, group dynamics and atmosphere and to assist the interviewer when there were questions. As such factors may influence the discussion, observations were recorded in field notes. A physiotherapist (A.N.) was present to provide health education to the participants after the interviews. Following each FDG, the research team convened to debrief, discuss observations and reflect on emerging impressions. These reflections informed subsequent data collection and were incorporated into the analytical process. A local Malawian data collector conducted all FDGs while the foreign researchers maintained an observational role throughout to mitigate the possible influence of their presence on participant engagement and data interpretation. Each session began with showing photographs of children with CP to establish a common understanding of the condition and assess participants' existing knowledge and terminology for the disability. The photographs illustrated different presentations of CP (athetoid, spastic and dyskinetic). No explanation was accompanied with the images, as the intention was to elicit participants' initial reaction and interpretations. This allowed the researchers to explore how participants described children with CP, the terms they used to refer to the condition and the perceptions regarding its possible causes. FGDs lasted between 40 and 80 min and were audio‐recorded after participant consent.

### 2.4 Data Analysis

Audio recordings were transcribed verbatim and translated into English by two independent translators to minimize translation bias. Data were analysed using Braun and Clarke's six‐step process of thematic analysis (TA) (Braun and Clarke 2021). The following TA steps are summarized below:

- Familiarization with the data
- Generating initial codes
- Searching for themes
- Reviewing themes
- Defining and naming themes
- Writing a research report

The analysis process was a hybrid inductive‐deductive approach, guided by the ICF framework underpinning the topic guide. Initially, two researchers (B.v.d.M. and V.v.S.) independently inductively coded transcripts using colour‐coding techniques, allowing themes to emerge directly from the data. As the coding process progressed and key topics became more defined, a more deductive approach was applied to organize the data within those emerging domains, while discrepancies were resolved through discussion until consensus was reached. Field notes from the observers' research diaries and post‐FGD team debriefs informed and enriched the analytical process. Data saturation was considered achieved when no new themes emerged from subsequent interviews.

Chat GTP‐5 by Open AI was used for language editing and paraphrasing to improve clarity and readability. The model did not generate novel scientific content or analyse data.

### 2.5 Ethical Considerations

Ethical approval was obtained from the Ethical Board (COMREC, P.08/22/3714). All participants were asked for consent before participation; if agreed, informed consent form was signed or, if unable to write, a thumbprint was used with an impartial witness present to verify the process. Subject information was provided in local languages, and participant anonymity was maintained throughout the study.

## 3 Results

### 3.1 Participant Characteristics

Eleven FGDs were conducted with a total of 68 participants across five villages and one MCV site. The interviews with the traditional leaders consisted of one to three persons and the interviews with the other stakeholders between eight and 12 participants. Village populations ranged from 900 to 3994 inhabitants. Participant distribution included traditional leaders; chiefs (*n* = 5) and counsellors (*n* = 9), religious leaders (*n* = 13), teachers (*n* = 17), traditional healers (*n* = 6), community health workers, HSAs (*n* = 13) and community health volunteers from MCV (*n* = 5).

### 3.2 Thematic Analysis Results

The following four major themes emerged from the analysis: (1) knowledge and beliefs surrounding CP, (2) perception of difficulties and their impact, (3) participation within the community and community response and (4) future facilitators to improve quality of life.

#### 3.2.1 Knowledge and Beliefs Surrounding CP

##### 3.2.1.1 Limited Disease Understanding and Terminology

Most participants recognized children with CP from photographs but lacked specific terminology to describe the condition. Due to the absence of specific terminology for CP in the local language, participants used descriptive or comparative language, using phrases such as ‘born with disability,’ ‘something went wrong at birth’ using phrases alluding to the causes of the condition, such as ‘born with disability,’ ‘something went wrong at birth’, but also labels directly describing the functional limitation, such as ‘lame person,’ or ‘crippled children.’ This variation in language across participants suggests differing underlying attitudes towards children with CP. It is well known that terminology that reduces a person to his or her functional limitation may be stigmatizing and reinforce social distance and exclusion.

The absence of a specific terminology also created a conceptual vacuum that was filled by familiar disease labels. Many participants incorrectly identified the condition as polio due to familiarity with this disease and similar mobility limitations.

*‘Normally we just call this condition polio for the fact that the doctors at the hospital do not tell us the actual name of the condition and because people are more familiar with polio, we just assume that the condition is polio as well.’* (Counsellor)

This mislabelling extended to causal beliefs, with some participants describing CP as a hereditary disease, believing that when infected with polio, there is a higher chance that down the line a child with CP will be born. This reflects how the absence of accurate medical framing allows existing disease narratives to shape community understanding of CP.

‘If the doctors were disclosing the name of this condition to the mothers, we would be in a position to know its name and how to take care of the baby, but the community is always in the dark and we literally have no idea on how best we can handle children with this condition and in the end, we just say this condition is stroke.’ (Teacher)

This quote illustrates how the absence of medical terminology has consequences for the child's care pathway. Difficulties in expressing themselves in the local language about medical conditions were thought to play an important role in the way people think about CP. It was mentioned that there was a lack of communication about the condition by health care professionals towards the respective families. In the absence of giving the condition a proper name, and using incorrect labels as ‘stroke’ and ‘polio,’ caregivers can be directed towards inappropriate care for their children.

##### 3.2.1.2 Perceived Causes of CP

Participants attributed CP to diverse causes categorized into the following five main areas: infectious diseases (polio, malaria, sexually transmitted diseases and TseTse fly infections), pregnancy‐related factors (teenage pregnancy, failed pregnancy termination, birth complications and malnutrition), cultural beliefs (witchcraft and family blamed for condition of child), mental health issues and other factors (domestic violence and hereditary causes).

###### 3.2.1.2.1 Pregnancy‐Related Factors

Several pregnancy and birth‐related causes were identified, though not all were medically accurate or stigmatizing. It was known that teenage pregnancy is a risk factor, with participants understanding that young mothers' bodies may not be fully prepared for childbirth, leading to complications that could result in CP. Birth complications such as lack of oxygen during birth, big babies and low birth weight were also correctly identified as potential causes. On the other hand, there were the ideas that women who attempted to terminate the pregnancy, but did not succeed, would affect the development of the unborn child, and as a result, the child would get CP. A belief that places moral blame directly on the mother and is likely to contribute to her social isolation and stigmatization in the community.

###### 3.2.1.2.2 Cultural Beliefs

Witchcraft emerged as a prominent explanatory model among participants. Many described CP as resulting from curses or spells that have been cast on the child or their child's family. These spells were thought to be imposed either by family relatives or by individuals outside the family Participants explained witchcraft accusations often arise in context of jealousy or wrongdoing like interpersonal conflict or perceived inequities in the distribution of resources within families or communities. Such explanatory models assign blame to the child's family for the child's condition and contribute to stigmatization and social isolation of affected families, as communities may distance themselves from families perceived to be entangled in witchcraft.

*‘This is now my personal experience, it might happen that a baby might be born normal but witches might cast a spell on the baby and all of a sudden the baby becomes disabled… so yes, there is that popular belief that witchcraft takes the share as one of the causes of CP.’* (Sheikh)

##### 3.2.1.3 Treatment Perceptions and Referral Knowledge

Opinions varied regarding CP treatability, ranging from belief in complete cure to acceptance of the condition as unchangeable. Most participants viewed hospital‐based medical treatment as the primary intervention, although some acknowledged roles for traditional medicine and religious healing when medical approaches failed.

Knowledge of referral pathways was inconsistent, with some participants uncertain about where to access services, NGOs or hospital and where in the hospital. Within the rural communities in Malawi, it is sometimes thought that referral letters from chiefs are required for accessing health care; uncertainty regarding the need for such letters in case of CP was also brought up during the interviews. This confusion potentially delayed appropriate care‐seeking.

#### 3.2.2 Perception of Difficulties and Their Impact on the Children and Their Caregivers

##### 3.2.2.1 Caregiver Challenges

Families with children with CP are facing a variety of difficulties according to the participants. The difficulties range from financial difficulties, lack of food and housing, to marital difficulties and abandonment of the child's mother, as well as stigma, social isolation and shame.

Financial difficulties were commonly named as a challenge for caregivers of children with CP. Participants described how caring for children with CP prevented caregivers, typically mothers, from engaging in income‐generating activities (e.g., work in the fields), leading to poverty and financial instability, often causing problems within the marriage. As a result, husbands are regularly abandoning their women and children, which in turn leads to even more financial difficulties for the women. This vicious cycle also brings difficulties in providing other basic needs as food, housing and clothes. In addition, lack of access to health care services was reported due to inability of these children to walk, leading to extra expensed for (public) transport, which affected accessibility.

*‘The presence of financial inactivity for both parents, brings about a lot of problems and marital tension. The husband may want to leave the wife and remarry somebody else. This will pile more misery in the life of both the mother and her child with CP.’* (Lay Leader)

Paradoxically, if the family of a child with CP manages to sustain a living and be financially stable, community members believe that the condition must be associated with the use of traditional medicine or other supernatural influences, indicating that the child was possibly being disabled on purpose for the sake of better business opportunities.

‘There are a lot of problems, if the family of the child with CP is financially stable or their business is progressing well, people around the community will be saying the child's disability is not by coincidence, the family used some charms or traditional medicine that caused the child to be to be disabled.’ (Primary School Teacher)

Another pervasive issue faced by the caregivers is stigma, both experienced and felt and social isolation. While caregivers experience most stigma within their own family or within the community, children are often mocked and stigmatized by their peers or in school. Comments such as ‘look at her, she has a crippled baby’ were commonly reported, leading to psychological distress and social exclusion, with mothers often remaining homebound to avoid public ridicule. This can have potential severe psychological effects on the parents as well as on the children.

‘At times these families who have children with CP suffer stereotyping, such things affect both the parent and the child either in a short term or long‐term point of view. The mother might get psychologically affected and start asking questions such as why me? For the child, he/she might get it tough to associate with their peers.’ (HSA)

##### 3.2.2.2 Challenges for Children With CP

Children faced physical limitations that restricted participation in daily activities and peer interactions. They cannot play with their peers, and they do need special equipment to move around, which often exposes them to mocking and discrimination. The children are often perceived as ‘difficult to handle’ by different community members, which makes people less willing to support the families. Educational barriers included lack of special‐needs schools and teachers, transportation difficulties and inadequate special needs materials. These limitations often resulted in children remaining at home without educational stimulation or social engagement. When enrolled at school they tend to fall behind in class due to cognitive impairments and learning difficulties, leading to difficulties following the class. This disrupts the learning process for other students can lead to tensions within the classroom and extra work for the teachers.

*‘There is always some form of stigma in the sense that when we register children with CP, the other learners grow in curiosity, they gather around that particular learner with CP and start asking questions that are mockery in nature. Some teachers do not pay any attention to learners with CP and such learners will feel neglected.’* (Primary School Teacher)

***‘**It requires a lot of time to make the children with CP grasp a concept you are teaching, and in most cases, the learners with CP are moody and they are difficult to give instructions to. You tell them to do something, but than they do something different.’* *(*Primary School Teacher)

##### 3.2.2.3 Community Perceptions of MCV Volunteers

Despite their supportive role, MCV volunteers faced negative community attitudes due to misconceptions about financial benefits. Many community members believed volunteers were misusing funds intended for children with CP, creating mistrust and impeding their effectiveness.

‘We receive a lot of insults from the communities, they think we get a lot of gifts or handouts that we were supposed to give to the children. This misconception is widely shared among the community members and sometimes it gets back to us in a negative way, our reputation is at stake, but we don't get discouraged like my friends have already said.’ (MCV Volunteer)

#### 3.2.3 Community Participation and Response

##### 3.2.3.1 Positive Inclusion Efforts

Community and religious leaders generally expressed positive attitudes towards including children with CP in community activities. Chiefs promoted ‘inclusive facilities’ and prioritized families with CP for aid distribution. The community leaders are aiming at creating a community sense of ‘togetherness,’ a sense of belonging together to make the community bounds stronger and to reduce discrimination. Religious leaders often viewed these children as ‘special gifts from God’ and sought to connect families with social services.

*‘Families with children with CP are always prioritized in all the community activities especially when there are provisions of distributing aid.’* (Chief)

##### 3.2.3.2 Barriers to Participation

Despite positive intentions, practical barriers limited meaningful participation. These included disruptions by children with CP during religious services because they would make noise or not sit still, lack of appropriate equipment (wheelchairs and adaptive seating) and limited community awareness about disability needs. Educational barriers were particularly prominent, with teachers lacking training and resources to accommodate children with special needs.

*‘Children with CP sometimes are a handful to handle and some members cannot handle it and start to complain that their prayer sessions going to be spoiled by the child's naggings and noise in the prayer house.’* (Sheikh)

##### 3.2.3.3 Healthcare Provider Perspectives

HSAs acknowledged limited training in disability management and relied primarily on personal experience when counselling families. Traditional healers reported fewer CP cases than previously, as most families now sought hospital care first. However, they continued providing counselling and spiritual support (‘to trust on god because he is the one who heals people not us’) while encouraging medical treatment.

#### 3.2.4 Future Facilitators to Improve Quality of Life

##### 3.2.4.1 Community Sensitization Needs

Participants emphasized community education about disabilities as the most critical intervention needed. They recognized that discrimination and social exclusion represented major barriers to health promotion and requested more inclusive activities and better cooperation between stakeholders.

##### 3.2.4.2 Healthcare System Strengthening

Stakeholders identified the health sector as the primary actor in organizing CP care but noted needs for clear guidelines, better training and improved referral pathways. They emphasized the importance of integrated approaches combining medical care, education, nutrition and psychosocial support.

*‘To be an integrated intervention on the care of these children with CP. Those involved in nutrition should play their part, those in the education sector should play their part as well.’* (MCV Volunteer)

##### 3.2.4.3 Resource and Support Needs

Participants highlighted needs for equipment provision (wheelchairs and adaptive devices), income‐generating activities for caregivers, improved transportation and enhanced communication between health workers and community leaders.

*‘If there was the possibility to introduce a lead or an informant who could be based in the community and this lead should be the focal point between the family and those special needs service providers. This so because most of the families who have children with CP do not know where to take their children to for help.’* (Church)

All the findings are summarized in Table 1.

**TABLE 1:** Key findings and implications for community‐based health promotion.

Theme	Key findings	Implications for practice/action
Knowledge and terminology	No local terms for CP exists; participants used stigmatizing labels such as ‘lame’ and ‘crippled’; CP frequently misidentified as polio or stroke	Develop culturally appropriate CP education materials in Chichewa and Chiyao; Spread those through community awareness programmes through for example radio and community dialogues to improve understanding of CP and address harmful beliefs. Training for health workers to communicate diagnosis clearly to families and communities
Causal beliefs	CP attributed to witchcraft, ‘wrongdoing’ and infectious diseases alongside some medically accurate causes; witchcraft beliefs assign blame to mothers and drive stigma. Financial stability paradoxically attributed to supernatural causes.	Community sensitization programmes like radio, drama play and community dialogues to target harmful, causal beliefs; Engage community stakeholders such as religious and traditional healers as agents of change given their influence on community norms and values.
Impact on the family: socio economic impact	Caring responsibilities prevent income generation, leading to poverty marital issues and abandonment of the mothers	Integrate income‐generating activities and social protection into CP care programmes; Address financial barriers to healthcare access including transport support.
Stigma and social exclusion	Some communities demonstrated supportive attitudes and inclusion efforts However many families experience stigma from family, peers and community Mothers remain home to avoid ridicule Children face exclusion and bullying in the communities and at school.	Community sensitization programmes targeting stigma and discrimination through radio and community dialogue. Engage community and religious leaders to promote rights‐based narratives. Train teachers in inclusive education.
Participation barriers	Despite positive intentions, practical barriers limit inclusion – lack of equipment, inadequate school resources, disruption during religious services.	Provide adaptive equipment; develop inclusive school programmes with trained special needs teachers; create disability‐friendly spaces in religious and community settings
Healthcare system gaps	HSAs lack training in disability management Referral pathways unclear Families unaware of available services	Strengthen HSA training in CP identification and referral Establish clear community‐based referral pathways with a designated community focal point
Community support structures	Religious and community leaders expressed willingness to support families MCV volunteers undermined by community mistrust	Leverage leaders as health promotion champions Improve transparency around volunteer roles and resources to rebuild community trust

## 4 Discussion

This study explores the perception, beliefs and attitudes of community stakeholders, namely: traditional leaders, religious leaders, educators, community health workers and traditional healers, towards children with CP and their caregivers in rural Malawi. The ICF model provides a useful theoretical framework for interpreting these findings (World Health Organization 2001). By conceptualizing disability across body functions, activity and participation, environmental factors and personal factors, it makes clear that the barriers identified in this study—stigma, witchcraft beliefs, financial hardship, participation restrictions and limited services—cannot be addressed through medical management alone (Santana et al. 2024). By exploring both barriers and opportunities for community‐based health promotion, the findings contribute to the development of locally acceptable strategies to reduce stigma, promote inclusion and strengthen support for families affected by CP. This is particularly relevant in a context where stigma and discrimination are widespread, community stakeholders’ perspectives remain largely unstudied and existing services are limited. The findings align with WHO's Global Report on Health Equity for Persons with Disabilities, which emphasizes the importance of addressing barriers faced by people with disabilities to achieve Sustainable Development Goals ensuring health for all (WHO 2022).

### 4.1 Knowledge Gaps and Health Promotion Opportunities

The limited understanding of CP aetiology and terminology among stakeholders represents both a challenge and an opportunity for health promotion (Sage et al. 2023). Following Green et al. (1993), the identified misconceptions can be categorized into the following three groups: (1) harmful beliefs requiring immediate attention, (2) potentially beneficial misconceptions that can be leveraged for health education and (3) neutral misconceptions of lower priority (Green et al. 1993). Harmful beliefs, particularly those linking CP to witchcraft and cultural practices, demand urgent attention as they perpetuate stigma and social exclusion. Pregnancy‐related misconceptions present a mixed picture but offer potential pathways for improving antenatal care attendance and CP prevention strategies. Less harmful misconceptions, such as those concerning various infectious diseases, warrant lower intervention priority.

The community's general recognition of CP as a distinct condition, combined with stakeholders' expressed willingness to learn, indicates strong potential for effective interventions like radio drama, community dialogues and health education peer groups in the community to improve knowledge and address harmful beliefs (Bilali 2022; Bandopadhay et al. 2021). These findings align with existing literature on sociocultural factors affecting families of children with intellectual disabilities and barriers to caring for children with CP in similar settings (Pitten 2018; Skinner and Weisner 2007; Rachamose and Harvey 2025).

The absence of local language terminology for CP highlights the need for culturally appropriate health communication strategies in Chichewa, the main language in Malawi and local language like Chiyao, which is widely spoken in Mangochi. Developing locally meaningful descriptions and educational materials, could improve community understanding and reduce reliance on stigmatizing terms. It would also help support healthcare workers in communicating diagnoses clearly to families. Crucially, the development and dissemination of such materials should actively involve community stakeholders—including traditional leaders, religious leaders and community health workers—given their established influence on community norms and their potential role as agents of change (Lemmi et al. 2015; Pitten 2018; Kang‐Yi et al. 2018). As the findings of this study demonstrate, the success of any health promotion strategy in this context depends heavily on the engagement and buy‐in of these key community figures.

### 4.2 Socioeconomic Determinants of Health

The prominence of financial difficulties as a primary challenge aligns with broader literature on disability and poverty in LMICs (Green 2007; Groce et al. 2011). The cycle of poverty described by participants—where caring responsibilities prevent income generation, leading to family instability and abandonment—demonstrates how CP affects entire family systems and communities (Dambi et al. 2015). This finding emphasizes the need for health interventions to also address socioeconomic determinants rather than focusing solely on medical management. In Bangladesh, caregivers received micro finance loans, which improved the health‐related quality of life of the children and the social capital of the families (Al Imam et al. 2023).

The association between caring for a child with disability and financial instability has been documented across multiple African contexts, including Tanzania, South Africa and Zimbabwe (Pretorius and Steadman 2018; McNally and Mannan 2013; Paget et al. 2015; Dambi et al. 2015). However, this study reveals a particularly striking cultural dynamic in rural Malawi, where financial stability among families of children with CP was interpreted by community members as evidence that the disability had been deliberately caused through the use of traditional medicine or charms for financial gain. This belief represents an extension of the witchcraft explanatory model discussed above, where disability is rarely perceived as coincidental but rather as the intentional result of someone's actions. The result is a double bind for affected families—poverty is seen as the expected and deserved consequence of having a child with CP, while financial resilience is viewed with suspicion and moral judgement. In either situation, families cannot escape stigma. This finding has direct implications for intervention design. Income‐generating activities and financial support programmes—which are widely recommended for caregivers of children with disabilities in LMICs—must in this context be accompanied by targeted community sensitization. Without addressing the underlying explanatory beliefs, improved family finances may paradoxically increase rather than reduce stigma and social isolation, undermining the very outcomes such interventions seek to achieve.

### 4.3 Stigma and Social Participation

While stakeholders expressed generally positive attitudes towards inclusion, the persistence of stigma and discrimination indicates a gap between stated values and lived experiences of families. For instance, stakeholders intend to welcome the families in their mosques and churches, but only if they behave ‘normal.’ Similar stories were shared in the educational context, where children were often described as ‘difficult’ or ‘moody.’ Families reported experiencing multiple forms of enacted stigma (Gray 2002a) including peer bullying, verbal insults directed at mothers, stereotyping and attributing disability to witchcraft. In examining previous studies from Africa it becomes apparent that one reason why caregivers are stereotyped is because of the perception of disabilities. In Uganda and Kenya the perception that disability is the work of evil spirits or a curse or god's punishment results in children with a disability being hidden (Gona et al. 2011). This discrepancy suggests that health promotion interventions must address both explicit discrimination and implicit biases that currently limit meaningful community participation (Adungna et al. 2020). Additionally, mothers reported staying at home to avoid public ridicule and psychological distress, which reflects a form of felt stigma (Gray 2002a).

### 4.4 Healthcare System Integration

The stakeholders' emphasis on medical treatment as the primary intervention, while acknowledging limitations in current services, suggests opportunities for strengthening health system capacity. The interest in integrated approaches, combining medical care, education and psychosocial support, would improve access to care and could benefit also the caregiver. This is in line with the WHO report on global disability to integrated health services with an emphasis on primary care and essential public health functions (Sage et al. 2023; WHO 2022).

The limited training among HSAs and teachers represents a missed opportunity for community‐based health promotion in Malawi, given their trusted positions and regular community contact. Targeted training programmes could significantly enhance early identification, referral and ongoing support for children with CP. The negative perceptions of MCV volunteers despite their supportive role illustrate how community mistrust can undermine well‐intentioned health promotion efforts. This finding highlights the importance of transparent communication and community engagement in program implementation.

### 4.5 Strengths and Limitations

This is the first study assessing community stakeholder perceptions, beliefs and attitudes towards children with cerebral palsy and caregivers in Malawi. Interviews were conducted in Chichewa and Chiyao in familiar environments, with two independent translators minimizing translation bias. Findings have broader relevance for community‐based CP care in similar low‐resource contexts, with themes of knowledge gaps, financial constraints, stigma and healthcare limitations resonating across SSA (scior 2011; Dambi et al. 2015). However, several limitations should be acknowledged. Social desirability bias may have influenced responses given participants' awareness of researchers' intent to establish a CP program. Additionally, despite efforts to minimize translation bias, the translation from Chichewa and Chiyao to English may have resulted in some loss of cultural meaning. Furthermore, the group discussion format may have limited the depth of data on sensitive topics such as witchcraft, as participants may have been less willing to speak openly in the presence of peers, particularly community and religious leaders. Future studies using individual interviews may be better placed to explore these themes in greater depth.

## 5 Conclusions

This study explored the perception, beliefs and attitudes of key community stakeholders towards children with CP and their caregivers' in rural Malawi. These results identified both barriers and opportunities for community‐based health promotion. Findings revealed significant knowledge gaps, stigmatizing terminology and harmful causal beliefs—including witchcraft—that contribute to social exclusion and marginalization of affected families. Financial hardship, inadequate healthcare infrastructure and limited educational support further compound these challenges. Positive community attitudes provide a strong foundation for inclusive program development. Addressing these challenges requires culturally appropriate health communication in local languages, targeted community sensitization, strengthened health worker training and integrating medical and socioeconomic needs such as by income‐generating cooperatives for caregivers. Integrating community stakeholder perspectives into health promotion planning is critical for achieving health equity for children with disabilities in resource‐limited settings.

These findings inform disability‐inclusive health promotion in low‐resource settings. Future research should examine intervention effectiveness in increasing knowledge, reducing stigma and decreasing participation barriers.

## Author Contributions

**Bente van der Meijden:** conceptualization, writing – original draft, validation, data curation. **Victoria von Salmuth:** investigation, formal analysis, writing – review and editing. **Alpheus Njewa:** investigation, conceptualization. **Alinafe Chisulanda Kusate:** writing – review and editing. **Ria Reis:** supervision, writing – review and editing. **Wieger Voskuijl:** writing – review and editing, supervision.

## Funding

This research did not receive any specific grant from funding agencies in the public, commercial, or not‐for‐profit sectors.

## Ethics Statement

Ethical approval for this study was obtained from the COMREC, e.g., Malawian Medical Ethical Board (Approval No. [P.08/22/3714]). All methods were carried out in accordance with relevant guidelines and regulations.

## Consent

Written informed consent was obtained from all participants.

## Data Availability

The datasets used and/or analysed during the current study are available from the corresponding author on reasonable request.

## References

[cch70361-bib-0001] Adnams, C. M. 2010. “Perspectives of Intellectual Disability in South Africa: Epidemiology, Policy, Services for Children and Adults.” Current Opinion in Psychiatry 23, no. 5: 436–440. 10.1097/YCO.0b013e32833cf200. 20683179

[cch70361-bib-0002] Adungna, M. , F. Nabbouh , S. Shehata , and S. Ghahari . 2020. “Barriers and Facilitators to Healthcare Access for Children With Disabilities in Low and Middle Income Sub‐Saharan African Countries: A Scoping Review.” BMC Health Services Research 20, no. 1: 15. 10.1186/s12913-019-4822-6. PMC6945633 31906927

[cch70361-bib-0003] Al Imam, M. H. , I. Jahan , M. C. Das , S. M. K. Bashar , and G. Khandakar . 2023. “Supporting People in Extreme Poverty With Rehabilitation and Therapy (SUPPORT CP): A Trial Among Families of Children With Cerebral Palsy in Bangladesh.” Developmental Medicine and Child Neurology 65, no. 6: 773–782. 10.1111/dmcn.15445. 36335570

[cch70361-bib-0004] Bandopadhay, N. , G. B. Woelk , M. P. Kieffer , and D. Mpofu . 2021. “Project ACCLAIM: Intervention Effect on Community Knowledge, Attitudes and Beliefs of Maternal and Child Health and HIV/AIDS in Eswatini, Uganda and Zimbabwe.” AIDS and Behavior 25: 2400–2409. 10.1007/s10461-021-03210-7. PMC8222038 33661426

[cch70361-bib-0005] Bax, M. , M. Goldstein , P. Rosenbauw , et al. 2005. “Proposed Definition and Classification of Cerebral Palsy.” Developmental Medicine and Child Neurology 47, no. 8: 571–576. 10.1017/s001216220500112x. 16108461

[cch70361-bib-0006] Bilali, R. 2022. “Fighting Violent Extremism With Narrative Intervention: Evidence From a Field Experiment in West Africa.” Psychological Science 33, no. 2: 184–195. 10.1177/09567976211031895. 34990306

[cch70361-bib-0007] Braun, V. , and V. Clarke . 2021. Thematic Analysis: A Practical Guide. Sage.

[cch70361-bib-0008] Dambi, J. M. , J. Jelsma , and T. Mlambo . 2015. “Caring for a Child With Cerebral Palsy: The Experience of Zimbabwean Mothers.” African Journal of Disability 4, no. 1: 168. 10.4102/ajod.v4i1.168. PMC5433478 28730030

[cch70361-bib-0009] Donald, K. A. , P. Samia , A. Kakooza‐Mwesinge , and D. Bearden . 2014. “Pediatric Cerebral Palsy in Africa: A Systematic Review.” Seminars in Pediatric Neurology 21: 30–35. 10.1177/0883073814549245. 24655402

[cch70361-bib-0010] Eide, A. H. 2003. “Living Conditions Among People With Disabilities in Malawi: A National Representative Study.” Published online 2003.

[cch70361-bib-0011] Gona, J. K. , V. Mung'ala‐Odera , C. R. Newton , and S. Hartley . 2011. “Caring for Children With Disabilities in Kilifi, Kenya: What Is the Carer's Experience?” Child: Care, Health and Development 37, no. 2: 175–183. 10.1111/j.1365-2214.2010.01124.x. PMC3072473 20645990

[cch70361-bib-0012] Gray, A. 2002a. “Stigma in Psychiatry.” Journal of the Royal Society of Medicine 95, no. 2: 72–76. 10.1258/jrsm.95.2.72. PMC1279314 11823548

[cch70361-bib-0013] Gray, D. E. 2002b. ““Everybody Just Freezes. Everybody Is Just Embarrassed”: Felt and Enacted Stigma Among Parents of Children With High Functioning Autism.” Sociology of Health & Illness 24, no. 6: 734–749. 10.1111/1467-9566.00316.

[cch70361-bib-0014] Green, C. , A. Jurg , and A. Dgedge . 1993. “Sexually Transmitted Diseases, AIDS and Traditional Healers in Mozambique.” Medical Anthropology 15: 261–281. 10.1080/01459740.1993.9966094 8114622

[cch70361-bib-0015] Green, S. E. 2007. “We're Tired, Not Sad: Benefits and Burdens of Mothering a Child With a Disability.” Social Science & Medicine 64, no. 1: 150–163. 10.1016/j.socscimed.2006.08.025. 17014946

[cch70361-bib-0016] Groce, N. , G. Kembhavi , S. Wirz , and R. Lang . 2011. “Poverty and Disability – A Critical Review of the Literature in Low and Middle‐Income Countries.” 10.2139/ssrn.3398431.

[cch70361-bib-0017] Imam, A. , I. Jahan , M. Das , M. Muhit , and N. Badawi . 2021. “Population‐Based Surveillance of Children With Cerebral Palsy Enables Early Diagnosis and Intervention.” Developmental Medicine and Child Neurology 63: 883–884. 10.1111/dmcn.14861. 33719074

[cch70361-bib-0018] Kang‐Yi, C. D. , R. R. Grinker , R. Beidas , et al. 2018. “Influence of Community‐Level Cultural Beliefs About Autism on Families' and Professionals' Care for Children.” Transcultural Psychiatry 55, no. 5: 623–647. 10.1177/1363461518779831. PMC7008392 29972327

[cch70361-bib-0019] Katangwe‐Chirwa, T. J. , I. Jahan , A. Chitedze , T. Mankhoka , A. Mughogho , and The Malawi Cerebral Palsy Register . 2026. “Epidemiology of Cerebral Palsy in Malawi.” Developmental Medicine and Child Neurology 68: 1334–1335. 10.1111/dmcn.70174. 40836484

[cch70361-bib-0020] Lassetter, J. H. , B. L. Mandleco , and O. S. Roper . 2007. “Family Photographs: Expressions of Parents Raising Children With Disabilities.” Qualitative Health Research 17, no. 4: 456–467. 10.1177/1049732306298804. 17416699

[cch70361-bib-0021] Lemmi, V. , L. Gibson , K. Blanchet , K. S. Kumar , S. Rath , and S. Hartley . 2015. “Community‐Based Rehabilitation for People With Disabilities in Low‐ and Middle‐Income Countries: A Systematic Review.” Campbell Systematic Reviews 11, no. 1: 1–177. 10.4073/csr.2015.15.

[cch70361-bib-0022] McNally, A. , and H. Mannan . 2013. “Perceptions of Caring for Children With Disabilities: Experiences From Moshi, Tanzania.” African Journal of Disability 2, no. 1: a21. 10.4102/ajod.v2i1.21. PMC5442575 28729981

[cch70361-bib-0023] Mitra, S. , A. Posarac , and B. C. Vick . 2011. “Disability and Poverty in Developing Countries. A Snapshot From the World Health Survey.” SP Discussion Paper. Social Protection and Labor, World Bank. 10.2139/ssrn.1908128.

[cch70361-bib-0024] Novak, I. , M. Hines , S. Goldsmith , and R. Barclay . 2012. “Clinical Prognostic Messages From a Systematic Review on Cerebral Palsy.” Pediatrics 130, no. 5: 1285–1312. 10.1542/peds.2012-0924. 23045562

[cch70361-bib-0025] Opendataforafrica . n.d. “World Data Atlas, Malawi, Mangochi, Demographics.” Retrieved September 17, 2025. https://opendataforafrica.org/atlas/Malawi/Mangochi/Population‐projected.

[cch70361-bib-0026] Paget, A. , M. Mallewa , D. Chinguo , C. Mahebere‐Chirambo , and M. Gladstone . 2015. ““It Means You Are Grounded” – Caregivers' Perspectives on the Rehabilitation of Children With Neurodisability in Malawi.” Disability and Rehabilitation 38, no. 3: 223–234. 10.3109/09638288.2015.1035458. PMC4720048 25879468

[cch70361-bib-0027] Pitten, K. 2018. “How Cultural Values Influence Diagnosis, Treatment and the Welfare of Families With an Autistic Child.” InSight: Rivier Academic Journal 4, no. 1.

[cch70361-bib-0028] Pretorius, C. , and J. Steadman . 2018. “Barriers and Facilitators to Caring for a Child With Cerebral Palsy in Rural Communities of the Western Cape, South Africa.” Child Care in Practice 24, no. 4: 413–430.

[cch70361-bib-0029] Rachamose, N. , and S. Harvey . 2025. “The Barriers to Caring for a Child Living With Cerebral Palsy (CP) in Rural Limpopo, South Africa.” Disabilities 5, no. 1: 11. 10.3390/disabilities5010011.

[cch70361-bib-0030] Sage, R. , G. Mashinichini , and C. Ravesloot . 2023. “Disability and Rural Health.” In The Oxford Encyclopedia of Public Health. Oxford University Press. 10.1093/acrefore/9780190632366.013.492.

[cch70361-bib-0031] Santana, C. A. , P. Rosenbauw , J. van der Kemp , and A. C. de Campos . 2024. “Looking Beyond Body Structure and Function: ICF Foci and Who Is Being Assessed in Research About Adolescents and Young Adults With Cerebral Palsy—A Scoping Review.” International Journal of Environmental Research and Public Health 21, no. 6: 670. 10.3390/ijerph21060670. PMC11203594 38928917

[cch70361-bib-0032] scior, K. 2011. “Public Awareness, Attitudes and Beliefs Regarding Intellectual Disability: A Systematic Review.” Research in Developmental Disabilities 32, no. 6: 2164–2182. 10.1016/j.ridd.2011.07.005 21798712

[cch70361-bib-0033] Skinner, D. , and T. S. Weisner . 2007. “Sociocultural Studies of Families of Children With Intellectual Disabilities.” Mental Retardation and Developmental Disabilities Research Reviews 13: 302–312. 10.1002/mrdd.20170 17979204

[cch70361-bib-0034] Tataryn, M. , S. Polack , L. Chokotho , et al. 2017. “Childhood Disability in Malawi: A Population Based Assessment Using the Key Informant Method.” BMC Pediatrics 17, no. 1: 198. 10.1186/s12887-017-0948. PMC5704595 29179740

[cch70361-bib-0035] van der Meijden, B. , A. C. Kusate , A. Njewa , and W. Voskuijl . 2026. “Psychometric Properties and Validation of the Malawian Cerebral Palsy Quality of Life‐Child (CP‐QoL) Questionnaire for Primary Care Givers.” Malawi Medical Journal 38, no. 2: 87–93. 10.4314/mmj.v38i2.5. PMC13459002 42597164

[cch70361-bib-0036] van der Meijden, B. , H. J. Wedda , A. Njewa , A. C. Kusate , R. Reis , and W. Voskuijl . 2026. “Caring for a Child with Cerebral Palsy in Rural Malawi: A Mixed‐Methods Study of Caregiver Experiences and Support Needs.” Developmental Medicine and Child Neurology Published online in press. 10.1111/dmcn.70502 42748405

[cch70361-bib-0037] WHO . 2022. “Global Report on Health Equity for Persons With a Disability.” https://www.who.int/teams/noncommunicable‐diseases/sensory‐functions‐disability‐and‐rehabilitation/global‐report‐on‐health‐equity‐for‐persons‐with‐disabilities.

[cch70361-bib-0038] World Health Organization . 2001. “International Classification of Functioning, Disability and Health (ICF).” Who. https://www.who.int/classifications/icf/en/.

